# Attention-Based Multi-Objective Control for Morphing Aircraft

**DOI:** 10.3390/biomimetics10050280

**Published:** 2025-04-30

**Authors:** Qien Fu, Changyin Sun

**Affiliations:** 1 School of Automation, Southeast University, Nanjing 210096, China; cysun@seu.edu.cn; 2 School of Artificial Intelligence, Anhui University, Hefei 230601, China

**Keywords:** morphing aircraft, multi-objective control, optimal control, pseudospectral methods, attention mechanism

## Abstract

This paper proposes a learning-based joint morphing and flight control framework for avian-inspired morphing aircraft. Firstly, a novel multi-objective multi-phase optimal control problem is formulated to synthesize the comprehensive flight missions, incorporating additional requirements such as fuel consumption, maneuverability, and agility of the morphing aircraft. Subsequently, an auxiliary problem, employing ϵ-constraint and augmented state methods, is introduced to yield a finite and locally Lipschitz continuous value function, which facilitates the construction of a neural network controller. Furthermore, a multi-phase pseudospectral method is derived to discretize the auxiliary problem and formulate the corresponding nonlinear programming problem, where open loop optimal solutions of the multi-task flight mission are generated. Finally, a learning-based feedback controller is established using data from the open loop solutions, where a temporal masked attention mechanism is developed to extract information from sequential data more efficiently. Simulation results demonstrate that the designed attention module in the learning scheme yields a significant 53.5% reduction in test loss compared to the baseline model. Additionally, the proposed learning-based joint morphing and flight controller achieves a 37.6% improvement in average tracking performance over the fixed wing configuration, while also satisfying performance requirements for fuel consumption, maneuverability, and agility.

## 1. Introduction

Morphing aircraft have aroused increasing interest in the aeronautic and aerospace fields for their shape transformation ability during the flight procedure. Inspired by birds, the desired aerodynamic profile can be obtained for various flight conditions or tasks by morphing the wings or tails of the aircraft [1,2]. For instance, drones approximating northern goshawk are capable of extending their wings in aggressive flight to increase the maneuverability and agility, and tucking the wings and tails to reduce the power consumption in cruise flight [3]. Motivated by the albatross’ capacity for long-distance soaring flight without flapping, a variable camber wing is designed for aircraft to maintain optimal cruise efficiency [4]. Unmanned aerial vehicles mimicking the movements of the great black-backed gull possess wings with two morphing degrees-of-freedom and tails with three morphing degrees-of-freedom, which increase the maneuverability and controllability without traditional ailerons and elevators [5]. In [6], a non-flapping unmanned aerial vehicle is designed following the biological construction of great black-backed gulls’ feathers and bones, achieving effective wing deformation. In addition to birds, the structure of honeycombs also informs the design of flexible morphing aircraft, valued for their lightweight and high out-of-plane stiffness [7]. The adaptability and flexibility afforded by bio-inspired design principles, combined with advances in aircraft manufacturing, make morphing aircraft well suited for flight missions with comprehensive tasks including trajectory tracking [8], obstacle avoidance [9], and perching maneuvers [10].

While the morphing ability provides benefits in complicated flight conditions, additional uncertainties and nonlinearities caused by variations in the aerodynamic parameters, inertia, and moment also bring challenges to the flight stability and safety. In recent years, studies on flight control of morphing aircraft are generally split into two domains, which are separated control and coordinated control. Given that the varying aerodynamic coefficients influence the dynamics in a non-affine manner, most investigations assume predefined time-dependent morphing signals and design the flight control system subsequently. Linear parameter varying (LPV) models with gain-scheduled controllers are frequently utilized to handle the nonlinear dynamics during the morphing procedure [11,12,13,14]. In [15], an input–output pseudolinearization technique is conducted on sweep angle morphing aircraft to alleviate the cumbersome computation in nonlinear dynamic inversion. Adaptive backstepping control methods are also applied to track the altitudes, airspeeds, and attitudes [16,17]. Furthermore, sliding model control is exploited for wing morphing aircraft to maintain fuel-saving flight and enhance longitudinal maneuverability simultaneously [18,19,20]. With regard to improving the robustness in the morphing phase, neural networks including radial basis function (RBF) networks [21,22] and recurrent neural networks (RNNs) [23] are adopted with nonlinear observers to estimate the uncertainties brought by the shape changes. However, these works focus on the flight performance under the disturbances of shape transformation, where benefits of the morphing mechanism itself are ignored.

The objective of morphing is usually to optimize specific aerodynamic performance parameters of the aircraft, such as the lift coefficient, the drag coefficient, and the lift-to-drag ratio [24,25]. Ideal wing shapes can be calculated via shape optimization techniques [26,27], and reinforcement learning algorithms like Q-learning [28,29] and proximal policy optimization (PPO) [30] are adopted to generate more efficient airfoils. Nevertheless, tracking the desired shape accurately and instantaneously is a challenging task due to the inconsistency and uncertainty of the flight conditions [31]. In [32], an independent morphing decision module is designed with an anti-disturbance mechanism, where nonlinear optimization methods are utilized to minimize the engine thrust and decrease fuel consumption in cruise flight. Data-driven methods are also applied widely for morphing control to alleviate the complex environment and actuator modeling [33,34]. In [35], a morphing vehicle in the form of a rectangular parallelopiped is designed where the flight-condition-related optimal shape is tracked using actor–critic methods. Such framework is extended to ellipsoid-shaped aircraft with shape memory alloy (SMA) as morphing actuators, where the optimal morphing policy is generated by Q-learning [36]. The hysteresis characteristics of SMA actuators are investigated in [37] and a corresponding deep reinforcement learning morphing controller is developed with the soft actor–critic technique. In [38], macro-fiber-composites-based multifunctional airfoils are designed and the trailing edge is morphed using the PPO algorithm. Although a growing body of research on novel morphing control methods have been carried out, incorporating real-time morphing into the flight control loop is still a pivotal task.

The majority of works on simultaneous morphing and flight control follow a divided framework, where the morphing command is generated according to the flight condition or task, and the flight controller is designed or adapted subsequently to maintain the tracking performance with the morphing signal. In [39], the sweep angles of tandem wings are changed to decrease the additional inertia forces and moments without producing trim drag, and an LPV-based gain scheduled LQR controller is adopted to guarantee the stability and flying performance during the transition process. In [40], the reference area and span of telescopic wings are decided online by solving nonlinear optimization problems with flight-phase-dependent piecewise objective functions, where the shape changes are treated as disturbances in the tracking control module with an adaptive dynamic surface controller. The effects of morphing are also considered as model uncertainties in [41] when designing a cascaded flight controller, and the basic controller further serves as the training environment for a dueling deep Q-learning-based morphing policy. A similar profile is proposed in [42] where an incremental backstepping maneuver controller is designed and used to create an environment for learning a swept wing morphing controller with the SAC algorithm. Nevertheless, taking the morphing process as an external signal or disturbance cannot involve the coupling interaction between the shape transformations and flight dynamics in the controller design sufficiently. Moreover, generating morphing law via reinforcement learning methods requires vast amount simulations of flight tests in different conditions, and suffers from the trade-off between exploration and exploitation.

As mentioned above, morphing the wings or tails during the flight procedure adapts aerodynamic coefficients in real time, which makes morphing aircraft more capable of accomplishing tasks with multiple and time-varying targets than traditional aircraft. The multi-objective optimal control scheme has been applied in flight control for various types of aerial vehicles to satisfy comprehensive performance requirements such as reducing the control allocation error and fuel consumption concurrently [43,44,45,46]. However, multi-task flight for morphing aircraft has not been investigated in much detail. Pareto-based multi-objective optimization techniques are utilized for glide trajectory optimization of hypersonic morphing aircraft with a limited two-stage morphing capability, where the optimal ballistic parameters and morphing timing are calculated to obtain a maximum range of trajectory and a minimum absorbed heat of leading edge simultaneously [47]. This framework is extended to re-entry trajectory optimization of telescopic wing morphing aircraft and the morphing rate is selected as the decision variable [48]. Furthermore, an overall flight procedure is composed of different stages including climbing, cruise, landing, and so forth, where the multi-phase control problem needs to be solved to optimize specific performance for individual phases [49]. While the multi-phase optimal control scheme has been exploited for conventional aircraft such as space-plane-based launch vehicles [50,51] and innovative control effector tailless aircraft [52], how to improve the flight performance using the morphing ability in integrated missions still remains an open problem.

Motivated by the discussed investigations, in this paper we focus on flight scenarios with multiple and varying tasks, and propose the learning-based morphing and flight control framework. The contributions are as follows. Firstly, we model the comprehensive flight missions of morphing aircraft as a general multi-objective multi-phase optimal control problem where the morphing command is incorporated into control values. Specifically, a type of wing sweep morphing aircraft and an aggressive-cruise synthesized task are selected to demonstrate the developed problem in detail. Subsequently, we transform the original optimal control problem into an auxiliary problem with ϵ-constraint and augment state methods to admit a finite and locally Lipschitz continuous value function. Such problem transformation establishes connections between the open loop solutions and the optimal feedback control rule, and serves as the theoretical foundation of the following neural network-based closed loop controller. Furthermore, we discretize the auxiliary problem in multiple time phases and formulate the corresponding nonlinear programming (NLP) task based on pseudospectral methods, from which open loop solutions of the optimal control problem are generated by nonlinear optimization techniques. Finally, we construct the closed loop control net to learn a feedback controller using data from the open loop solutions, where a temporal masked attention mechanism is developed to extract information from sequence data more efficiently. Simulations are conducted to validate the proposed learning-based joint morphing and flight control framework.

The rest of this paper is organized as follows. In Section 2, we model the dynamics of the morphing aircraft and formulate the multi-objective multi-phase optimal control problem. In Section 3, we propose the learning-based coordinated flight and morphing control framework in three parts, which are problem transformation, open loop solutions generation, and closed loop controller learning. In Section 4, we validate the proposed method with simulations. In Section 5, we summarize this work.

## 2. Problem Formulation

In this section, we firstly construct the dynamic model of the morphing aircraft, and then formulate the joint morphing and flight control task as a multi-phase multi-objective optimal tracking problem for nonlinear systems.

**Figure 1:**
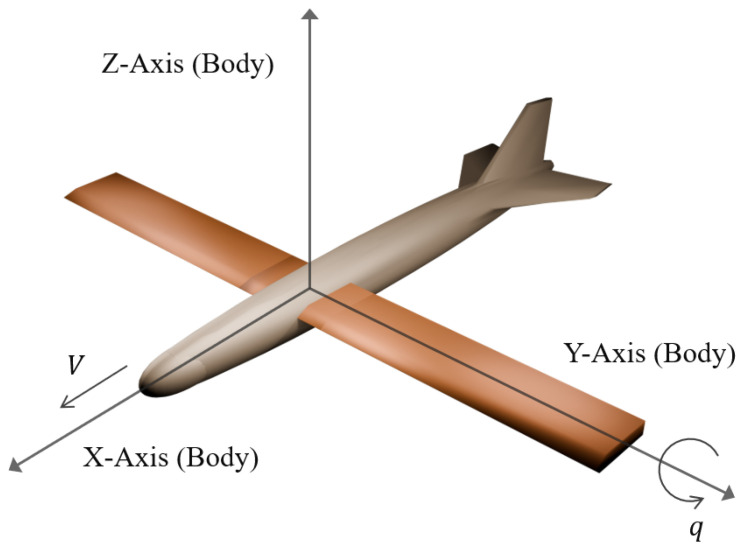
Illustration of the wing sweep morphing aircraft. Wings are extended with sweep angle ψ=0.

**Figure 2:**
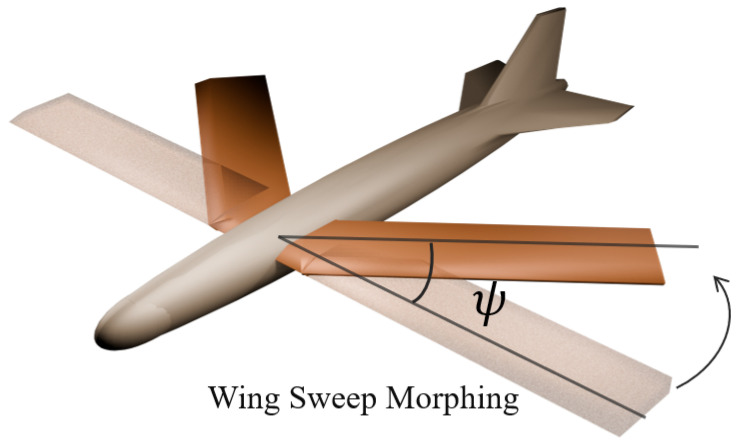
Illustration of the wing sweep morphing aircraft. Wings are tucked with sweep angle ψ=40deg.

Without loss of generality, we consider the longitudinal motion of a wing sweep morphing aircraft. In Figure 1 and Figure 2, the 3D model of the morphing aircraft and the morphing procedure are illustrated. Since the rate of morphing is relatively small compared to the flight speed, in the dynamics we assume that the terms related to the time derivative of the geometry are negligible [53]. The longitudinal dynamics are given as

(1) $$\begin{array}{cc} \dot{V} & =\frac{1}{m}\left(T\cos\alpha -D-mg\sin(\theta -\alpha )\right)\\ \dot{\alpha } & =q-\frac{1}{mV}\left(T\sin\alpha +L-mg\cos(\theta -\alpha )\right)\\ \dot{q} & =\frac{M}{J_{y}}\\ \dot{\theta } & =q\\ \dot{h} & =V\sin\gamma \end{array}$$

where the states *V*, α, *q*, θ, and *h* denote the longitudinal velocity, angle of attack, pitch rate, pitch angle, and altitude, respectively. Additionally, *m*, *g*, and $J_{y}$ are the mass, gravity acceleration, and pitching moment of inertia. *L*, *D*, *M*, and *T* denote the lift, drag, pitch moment, and thrust. The sweep angle ψ varies from 0 to $\psi_{\max}$, and the geometry of the aircraft is determined by the normalized morphing parameter $\xi ≜\psi /\psi_{\max}$, which is considered as an additional input for the system. Then, aerodynamic forces and moments are calculated according to

(2) $$\begin{array}{cc} L & =\frac{1}{2}\rho V^{2}SC_{L}\left(\alpha ,\xi \right)\\ D & =\frac{1}{2}\rho V^{2}SC_{D}\left(\alpha ,\xi \right)\\ M & =\frac{1}{2}\rho V^{2}S\bar{c}C_{M}\left(\alpha ,\xi ,\delta_{e}\right) \end{array}$$

where ρ, *S*, $\bar{c}$, and $\delta_{e}$ are the air density, wing area, mean aerodynamic chord, and elevator input, respectively. For aircraft with continuous shape morphing, the aerodynamic coefficients have different values for each ξ.

The basic task of the flight control in this work is to track a given reference trajectory. Moreover, the ability of morphing facilitates the improvement of other performances. The aerodynamic coefficients can be adjusted through the shape morphing to achieve different objectives in various flight scenarios. For example, in the cruise flight procedure the power requirement

(3) P=TV

is desired to be reduced. For the aggressive flight task, we aim to increase the maneuverability and agility [3]

(4) $$p_{a}=\left[\begin{array}{c} {\ddot{x}}_{wf}\\ {\ddot{z}}_{wf}\\ \dot{q} \end{array}\right]=\left[\begin{array}{c} \frac{-D}{m}-g\sin\alpha \\ \frac{L}{m}-g\cos\alpha \\ \frac{M}{J_{y}} \end{array}\right]$$

which evaluates the control ability of aircraft to change the velocity and angular rate. Denote the reference flight trajectory as $h_{r}\left(t\right)$, and the multi-objective Lagrange form cost function for cruise flight procedure $t\in T_{c}$ is given by

(5) $$J_{c}=\left[\begin{array}{c} \int_{T_{c}}{∥h-h_{r}∥}_{2}^{2}dt\\ \int_{T_{c}}Pdt \end{array}\right]$$

which indicates that we aim to decrease the tracking error and the power consumption simultaneously. Similarly, the cost function for aggressive flight procedure $t\in T_{a}$ is given by

(6) $$J_{a}=\left[\begin{array}{c} \int_{T_{a}}{∥h-h_{r}∥}_{2}^{2}dt\\ \int_{T_{a}}-{∥p_{a}∥}_{2}^{2}dt \end{array}\right]$$

which represents the requirement of improving the tracking accuracy, maneuverability, and agility.

After constructing the dynamics and objectives, we can formulate the joint morphing and flight control problem into a general form. Taking the morphing parameter ξ as an additional input, the above constructed motion dynamics can be summarized in the nonlinear differential equation

(7) dx=f(x,u)dt

with the state $x={\left[V,\gamma ,q,\alpha ,h\right]}^{⊤}$ and input $u={\left[\xi ,T\right]}^{⊤}$. According to various requirements of different tasks, the flight procedure is split into $N_{p}$ phases by switching times $0=t_{0}<t_{1}<\cdots <t_{N_{p}}=t_{f}$. Additionally, we denote the information from the reference trajectory as $x_{r}=h_{r}$ to facilitate further generalization. Each phase has a vector-valued cost function $J_{n}\left(x\left(t_{n-1}:t_{n}\right),u\left(t_{n-1}:t_{n}\right),x_{r}\left(t_{n-1}:t_{n}\right)\right)$ with elements

(8) $$J_{n,i}=\int_{t_{n-1}}^{t_{n}}l_{n,i}\left(x\left(t\right),u\left(t\right),x_{r}\left(t\right)\right)dt$$

which is a Lagrange form scalar cost for i=1,⋯,M. Then, the multi-phase multi-objective cost function is given by

(9) $$J=\sum_{n=0}^{N_{p}-1}J_{n}={\left[\sum_{n=0}^{N_{p}-1}J_{n,1},\cdots ,\sum_{n=0}^{N_{p}-1}J_{n,M}\right]}^{⊤}$$

The optimal control problem over a multi-objective cost is defined in the Pareto sense.

The problem in this work is to find a closed loop control rule $u^{*}\left(x,x_{r},t\right)$ that minimizes the multi-phase multi-objective cost function in the Pareto sense.

## 3. Methods

In this section, we develop a learning-based closed loop control policy for the proposed multi-phase multi-objective optimal control problem. The framework of policy learning is composed of an offline data generation procedure and state feedback control network training. Highly accurate open loop solutions are generated as a database via direct methods, and online closed loop control is then realized according to the trained policy.

### 3.1. Scalarization of Optimal Tracking Problem

As the various objects usually conflict with each other in multi-objective optimizations, there exist multiple different Pareto optimal solutions, which compose the Pareto set. By imposing the preference a priori, a unique optimal solution is selected via scalarizing the original problem and solving the transformed single objective optimization. Among the scalarization methods, the ϵ-constraint method is preferred for its ability to find Pareto optimal solutions in nonconvex Pareto sets, and to describe the trade-off between different objectives straightforwardly.

Based on the ϵ-constraint method, we select the first element of the objective function, which evaluates the tracking error, as the major objective. Other cost functions are transformed into constraints. Since in this work the tasks of different phases have different objectives, the aspiration level for each phase should be selected separately. Therefore, we construct the constraints for subordinate cost functions as

(10) $$J_{n,i}\leq \epsilon_{n,i}{\bar{t}}_{n},\;i=2,\cdots ,M$$

where ${\bar{t}}_{n}=t_{n}-t_{n-1}$ denotes the sojourn time of phase *n*. Note that the major objective is same in each phase. Additionally, subordinate objectives are irrelevant to the reference trajectory. Then, the original optimal tracking problem is converted to the single objective form with integral constraints as

(11) $$\begin{array}{cc} \min_{u} & J_{1}=\int_{0}^{t_{f}}l_{1}\left(x\left(t\right),u\left(t\right),x_{r}\left(t\right)\right)dt\\ s.t.\; & \dot{x}=f\left(x,u\right)\\ \; & J_{n,i}=\int_{t_{n-1}}^{t_{n}}l_{n,i}\left(x\left(t\right),u\left(t\right)\right)\leq \epsilon_{n,i}{\bar{t}}_{n},\;n=1,\cdots ,N_{p},i=2,\cdots ,M \end{array}$$

To convert the integral constraints to ODE constraints, N×M augmented variables are introduced by

(12) $$y_{n,i}\left(t\right)=\int_{0}^{t}{\bar{l}}_{n,i}\left(x\left(t\right),u\left(t\right),x_{r}\left(t\right)\right)$$

where the piecewise non-zero function ${\bar{l}}_{n,i}\left(x\left(t\right),u\left(t\right),x_{r}\left(t\right)\right)$ is given by

(13) $${\bar{l}}_{n,i}\left(x\left(t\right),u\left(t\right)\right)=\left\{\begin{array}{c} l_{n,i}\left(x\left(t\right),u\left(t\right)\right),\;t_{n-1}\leq t\leq t_{n}\\ 0,\;\mathrm{otherwise} \end{array}\right.$$

Consequently, the multi-objective optimal tracking problem is further transformed into

(14) $$\begin{array}{cc} \min_{u} & J_{1}=\int_{0}^{t_{f}}l_{1}\left(x\left(t\right),u\left(t\right),x_{r}\left(t\right)\right)dt\\ s.t.\; & \dot{x}=f\left(x\left(t\right),u\left(t\right)\right)\\ \; & \dot{y}=\bar{l}\left(x\left(t\right),u\left(t\right)\right)\\ \; & g(y\left(t_{f}\right))\leq 0 \end{array}$$

where $y={\left[y_{1,2},\cdots ,y_{N,M}\right]}^{⊤}$, $\bar{l}\left(x,u\right)={\left[{\bar{l}}_{1,2}\left(x,u\right),\cdots ,{\bar{l}}_{N,M}\left(x,u\right)\right]}^{⊤}$ and

(15) $$g\left(y(t_{f})\right)={\left[y_{1,2}\left(t_{f}\right)-\epsilon_{1,2}{\bar{t}}_{1},\cdots ,y_{N,M}\left(t_{f}\right)-\epsilon_{N,M}{\bar{t}}_{N}\right]}^{⊤}$$

For a given state feedback control policy u(x,y), denote the value function as $V\left(t,x,y\right)=\int_{t}^{t_{f}}l_{1}\left(x\left(t\right),u\left(t\right),x_{r}\left(t\right)\right)dt$. Without consideration of the end state constraints, it is well known that the value function is the viscosity solution of the Hamilton–Jacobian–Bellman (HJB) partial differential equation

(16) $$\begin{array}{cc} -\nabla_{t}V & =H\left(t,x,y,\nabla_{x}V,\nabla_{y}V\right)\\ \; & =l_{1}\left(x,u,x_{r}\right)+\nabla_{x}V^{⊤}f\left(x,u\right)+\nabla_{y}V^{⊤}\bar{l}\left(x,u\right) \end{array}$$

To solve the optimal control rule in an open loop, a necessary condition contributed by Pontryagin’s Minimum Principle (PMP) is given by the state and costate equations

(17) $$\left\{\begin{array}{cc} \dot{x} & =\nabla_{p_{x}}H\left(t,x,y,p_{x},p_{y}\right)=f\left(x,u\right)\\ \dot{y} & =\nabla_{p_{y}}H\left(t,x,y,p_{x},p_{y}\right)=\bar{l}\left(x,u,x_{r}\right)\\ \dot{p_{x}} & =-\nabla_{x}H\left(t,x,y,p_{x},p_{y}\right)\\ \dot{p_{y}} & =-\nabla_{y}H\left(t,x,y,p_{x},p_{y}\right) \end{array}\right.$$

Provided that the solutions to the PMP are globally optimal and the HJB equations admit classical $C^{1}$ solutions, the relationship between PMP and dynamic programming approaches is given by

(18) $$\begin{array}{c} p_{x}\left(t\right)=\nabla_{x}V\left(t,x\left(t\right),y\left(t\right)\right)\\ p_{y}\left(t\right)=\nabla_{y}V\left(t,x\left(t\right),y\left(t\right)\right) \end{array}$$

However, when the problem is imposed with end state constraints, the value function is generally discontinuous without some specified controllability assumptions [54]. A direct mapping between the open loop solutions and feedback solutions is not available in this case. Following the analysis in [55], we can introduce an auxiliary optimal control problem where the major objective is also wrapped in an augmented state. This auxiliary problem admits a more regular value function. Take the augmented state as

(19) $$z\left(t\right)=\int_{0}^{t}l_{1}\left(x\left(t\right),u\left(t\right),x_{r}\left(t\right)\right)dt$$

and then the original cost function is converted equivalently into a Mayer form as $-z(t_{f})$. The auxiliary problem is constructed as

(20) $$\begin{array}{cc} \min_{u} & \left\{\max\left(-z\left(t_{f}\right),\max_{i,n}g_{i,n}\left(y(t_{f})\right)\right)\right\}\\ s.t.\; & \dot{x}=f\left(x\left(t\right),u\left(t\right)\right)\\ \; & \dot{y}=\bar{l}\left(x\left(t\right),u\left(t\right)\right)\\ \; & \dot{z}=l_{1}\left(x\left(t\right),u\left(t\right),x_{r}\left(t\right)\right) \end{array}$$

and the corresponding value function is denoted by w(t,x,y,z). Since all the dynamic functions are continuous with respect to *x* and *u* for almost every t∈[0,T], we have w(t,x,y,z), the unique $L^{1}$-viscosity solution of the following HJB equation [55]

(21) $$\begin{array}{cc} -\nabla_{t}w & =H(t,x,y,z,\nabla_{x}w,\nabla_{y}w,\nabla_{z}w)\\ \; & =\nabla_{x}w^{⊤}f\left(x,u\right)+\nabla_{y}w^{⊤}\bar{l}\left(x,u\right)+\nabla_{z}wl_{1}\left(x,u,x_{r}\right)\\ w\left(x\left(t_{f}\right),y\left(t_{f}\right),z\left(t_{f}\right),t_{f}\right) & =\max\left(z\left(t_{f}\right),\max_{i,n}g_{i,n}\left(y(t_{f})\right)\right) \end{array}$$

If $\left\{x^{*}\left(t\right),y^{*}\left(t\right),z^{*}\left(t\right),u^{*}\left(t\right)\right\}$ is an optimal solution of the auxiliary problem (20) with optimal value function $w^{*}\left(t,x^{*}\left(t\right),y^{*}\left(t\right),z^{*}\left(t\right),u^{*}\left(t\right)\right)\leq 0$, then $\left\{x^{*}\left(t\right),y^{*}\left(t\right),u^{*}\left(t\right)\right\}$ is an optimal solution of the end state constrained problem (14). Directly solving an optimal feedback control rule in analytical form from the HJB equation is generally difficult. Motivated by the deep learning optimal control framework in [56], we learn the feedback policy for the problem (14) based on the open loop solutions to the auxiliary problem (20).

### 3.2. Generate Open Loop Solutions

Approaches for open loop solutions to optimal control problems are divided into the indirect methods and the direct methods. Indirect methods aim to solve the boundary value problem brought by the PMP numerically. A main drawback of indirect methods is that it is not straightforward to obtain an initial guess of costates. On the other hand, direct methods discretize the original problem first and transcribe it into a nonlinear programming problem. In this section, we generate the open loop solutions based on pseudospectral methods, which are class of direct collocation methods with spectral convergence [57].

Consider the problem (20) with only Mayer term cost. Pseudospectral methods are based on the Stone–Weierstrass theorem, which declares that a continuous function defined on a closed interval can be approximated to any desired accuracy by a polynomial [58]. Although the problem is described in one phase, the dynamics for the augmented variables *y* are instinctively phase-wise continuous. Therefore, we divide the time domain $\left[t_{0},t_{f}\right]$ into $N_{p}$ time intervals, according to the $N_{p}$ phases appointed by the flight task. The states $x(t^{\left(n\right)})$ in *n*th interval $t^{\left(n\right)}\in \left[t_{n-1},t_{n}\right]$ are denoted by $x^{\left(n\right)}$, and similar notations are used as $y^{\left(n\right)}$, $z^{\left(n\right)}$, $u^{\left(n\right)}$, and $x_{r}^{\left(n\right)}$. Then, the dynamics of states in each interval are continuous.

Subsequently, the trajectory in each interval is approximated with Gaussian grids and Lagrange interpolation. In this work, we select Legendre–Gauss–Labatto (LGL) collocation points, since compared with Legendre–Gauss (LG) and Legendre–Gauss–Radau (LGR) points, there is no requirement of assigning additional weights for LGL points [58]. In the *n*th time interval, domain transformation is conducted as

(22) $$t^{\left(n\right)}=\frac{t_{n}-t_{n-1}}{2}\tau^{\left(n\right)}+\frac{t_{n}+t_{n-1}}{2}$$

where the actual time domain $t^{\left(n\right)}\in \left[t_{n-1},t_{n}\right]$ is mapped to the computational domain $\tau^{n}\in \left[-1,1\right]$. The inverse mapping is given by

(23) $$\tau^{\left(n\right)}=\frac{2}{t_{n}-t_{n-1}}t^{\left(n\right)}-\frac{t_{n}+t_{n-1}}{t_{n}-t_{n-1}}$$

In the transformed time domain, $N_{\mathrm{LGL}}^{\left(n\right)}$ LGL collocation points ${\left\{\tau_{j}^{\left(n\right)}\right\}}_{j=1}^{N_{\mathrm{LGL}}^{\left(n\right)}}$ are selected by the roots of ${\dot{P}}_{N_{\mathrm{LGL}}^{\left(n\right)}-1}\left(\tau^{\left(n\right)}\right)$ together with −1 and 1, where $P_{N}\left(\tau \right)$ is the *N*th order Legendre polynomial

(24) $$P_{N}\left(\tau \right)=\frac{1}{2^{N}N!}\frac{d^{N}}{d\tau^{N}}{\left(\tau^{2}-1\right)}^{N}$$

Denote variables at the collocation point $\tau_{j}^{n}$ as $x_{j}^{\left(n\right)}$, $y_{j}^{\left(n\right)}$, $z_{j}^{\left(n\right)}$, $u_{j}^{\left(n\right)}$, and $x_{r,j}^{\left(n\right)}$. The trajectory of state $x^{\left(n\right)}$ is then approximated using a series of Lagrange polynomials as

(25) $$x^{\left(n\right)}\left(\tau^{\left(n\right)}\right)\approx {\hat{x}}^{\left(n\right)}\left(\tau^{\left(n\right)}\right)=\sum_{j=1}^{N_{\mathrm{LGL}}^{\left(n\right)}}\lambda_{j}^{\left(n\right)}\left(\tau^{\left(n\right)}\right)x_{j}^{\left(n\right)}$$

where

(26) $$\lambda_{j}^{\left(n\right)}\left(\tau^{\left(n\right)}\right)=\prod_{i=1,i\neq j}^{N_{\mathrm{LGL}}^{\left(n\right)}}\frac{\tau^{\left(n\right)}-\tau_{i}^{\left(n\right)}}{\tau_{j}^{\left(n\right)}-\tau_{i}^{\left(n\right)}}$$

Note that this approximation is accurate at the collocation points, since the Lagrange polynimials have the property that

(27) $$\lambda_{j}^{\left(n\right)}\left(\tau_{i}^{\left(n\right)}\right)=\left\{\begin{array}{c} 1,i=j\\ 0,i\neq j \end{array}\right.$$

To transcribe the original ODE constraints into algebra constraints, we differentiate the trajectory at each LGL point as

(28) $${\left.\frac{d{\hat{x}}^{\left(n\right)}\left(\tau^{\left(n\right)}\right)}{d\tau^{\left(n\right)}}\right|}_{\tau^{\left(n\right)}=\tau_{m}^{\left(n\right)}}=\sum_{j=1}^{N_{\mathrm{LGL}}^{\left(n\right)}}{\left.\frac{d\lambda_{j}^{\left(n\right)}\left(\tau^{\left(n\right)}\right)}{d\tau^{\left(n\right)}}\right|}_{\tau^{\left(n\right)}=\tau_{m}^{\left(n\right)}}x_{j}^{n}=\sum_{j=1}^{N_{\mathrm{LGL}}^{\left(n\right)}}D_{mj}^{\left(n\right)}x_{j}^{\left(n\right)}$$

for $m=1,\cdots ,N_{\mathrm{LGL}}^{\left(n\right)}$. The differential coefficients $D_{mj}^{\left(n\right)}$ compose the $N_{\mathrm{LGL}}^{\left(n\right)}\times N_{\mathrm{LGL}}^{\left(n\right)}$ singular square Lobatto pseudospectral differentiation matrix. The state dynamics from (20) are then imposed to the LGL points in the computational time domain, which gives the constraints

(29) $$\sum_{j=1}^{N_{\mathrm{LGL}}^{\left(n\right)}}D_{mj}^{\left(n\right)}x_{j}^{n}-\frac{t_{n}-t_{n-1}}{2}f\left(x_{m}^{\left(n\right)},u_{m}^{\left(n\right)}\right)=0$$

Similarly, the differential constraints for the auxiliary variable $z^{\left(n\right)}$ are transformed as

(30) $$\sum_{j=1}^{N_{\mathrm{LGL}}^{\left(n\right)}}D_{mj}^{\left(n\right)}z_{j}^{n}-\frac{t_{n}-t_{n-1}}{2}l_{1}\left(x_{m}^{\left(n\right)},u_{m}^{\left(n\right)},x_{r,m}^{\left(n\right)}\right)=0$$

With regard to the augmented variables *y*, we note that in the *n*th time interval, only the variables with subscript *n* have nonzero dynamics. Therefore, the constraints corresponding to augmented variables are given by

(31) $$\begin{array}{cc} \; & \sum_{j=1}^{N_{\mathrm{LGL}}^{\left(n\right)}}D_{mj}^{\left(n\right)}y_{n,i,j}^{\left(n\right)}-\frac{t_{n}-t_{n-1}}{2}l_{n,i}\left(x_{m}^{\left(n\right)},u_{m}^{\left(n\right)}\right)=0\\ \; & \sum_{j=1}^{N_{\mathrm{LGL}}^{\left(n\right)}}D_{mj}^{\left(n\right)}y_{n^{\prime },i,j}^{\left(n\right)}=0\;\mathrm{for}\;\mathrm{all}\;n^{\prime }\neq n \end{array}$$

In addition to the differential constraints in each individual interval, connection conditions between intervals should be satisfied to guarantee the continuity of each variable. Since the LGL points include τ=−1 and τ=1, the last collocation point in the previous interval coincides with the first point in the next interval, which gives that

(32) $$\begin{array}{cc} x_{N_{\mathrm{LGL}}^{\left(n\right)}}^{\left(n\right)} & =x_{1}^{\left(n+1\right)}\\ y_{N_{\mathrm{LGL}}^{\left(n\right)}}^{\left(n\right)} & =y_{1}^{\left(n+1\right)}\\ z_{N_{\mathrm{LGL}}^{\left(n\right)}}^{\left(n\right)} & =z_{1}^{\left(n+1\right)}\\ u_{N_{\mathrm{LGL}}^{\left(n\right)}}^{\left(n\right)} & =u_{1}^{\left(n+1\right)} \end{array}$$

for $n=1,\cdots ,N_{p}-1$.

Summarizing the conditions from (29)–(32), we obtain the equality constraints

(33) $$C\left(X,U,X_{r}\right)=0$$

where the stacked variables

(34) $$\begin{array}{cc} X & ={\left[x_{1}^{{\left(1\right)}^{⊤}},\cdots ,x_{N_{\mathrm{LGL}}^{\left(N_{p}\right)}}^{{\left(N_{p}\right)}^{⊤}},y_{1}^{{\left(1\right)}^{⊤}},\cdots ,y_{N_{\mathrm{LGL}}^{\left(N_{p}\right)}}^{{\left(N_{p}\right)}^{⊤}},z_{1}^{\left(1\right)},\cdots ,z_{N_{\mathrm{LGL}}^{\left(N_{p}\right)}}^{\left(N_{p}\right)}\right]}^{⊤}\\ U & ={\left[u_{1}^{{\left(1\right)}^{⊤}},\cdots ,u_{N_{\mathrm{LGL}}^{\left(N_{p}\right)}}^{{\left(N_{p}\right)}^{⊤}}\right]}^{⊤}\\ X_{r} & ={\left[x_{r,1}^{{\left(1\right)}^{⊤}},\cdots ,x_{r,N_{\mathrm{LGL}}^{\left(N_{p}\right)}}^{{\left(N_{p}\right)}^{⊤}}\right]}^{⊤} \end{array}$$

contain all the states, control inputs, and reference trajectories at collocation points, respectively.

The cost function in (20) is straightforwardly transformed into

(35) $$J\left(X,U\right)=\max\left(z_{N_{\mathrm{LGL}}^{\left(N_{p}\right)}}^{\left(N_{p}\right)},\max_{i,n}g_{i,n}\left(y_{N_{\mathrm{LGL}}^{\left(N_{p}\right)}}^{\left(N_{p}\right)}\right)\right)$$

Finally, by selecting *X* and *U* as decision variables, the nonlinear programming problem approximating the original optimal control task is formulated as

(36) $$\begin{array}{cc} \; & \min_{X,U}J\left(X,U\right)\\ s.t.\; & C\left(X,U,X_{r}\right)=0 \end{array}$$

Solutions to this NLP problem serve as open loop solutions to the optimal control problem, and are further exploited to learn the closed loop control rule in the next section.

### 3.3. Learn Closed Loop Control Law

**Figure 3:**
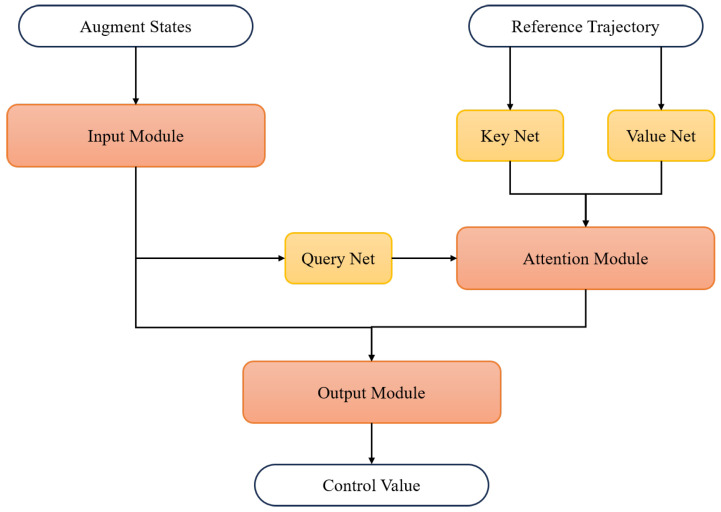
Overall structure of closed loop control net.

The transformed problem in (20) admits a finite and locally Lipschitz continuous value function. According to the relationship between the original multi-phase multi-objective optimal control problem and the auxiliary problem, we can learn the closed loop control law from the open loop solutions of the auxiliary problem. A supervised learning framework is adopted for generating the feedback control law, where the training datasets are collected using the pseudospectral method described in the previous section. The overall structure of the control net is depicted in Figure 3.

Before the training of the control net, the optimized collocated states and control values at LGL points are interpolated with Lagrange polynomials in (25) to obtain the full time trajectory $X^{\left(0:t_{f}\right)}$ and $U^{\left(0:t_{f}\right)}$. The control value at time *t* is generated by the closed loop control net in Figure 3, where the input includes current augment state $X^{\left(t\right)}$ and the reference trajectory $X_{r}^{\left(0:t_{f}\right)}$. The net is composed of three modules. Firstly, the input module extracts features from the current state by stacked fully connected layers with residual connections. Then, temporal information in the reference trajectory is exploited using an attention mechanism. Finally, outputs of the input module and attention module are concatenated and sent to the output module, which has a similar structure to the input module, to generate the online control value. Normalization of *X* and *U* is necessary to ensure that the neural network training is stable and converges efficiently.

**Figure 4:**
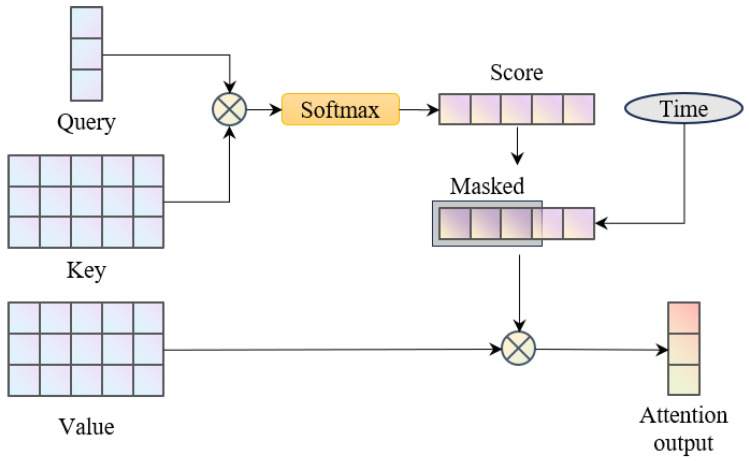
Illustration of the attention module.

Details of the attention module are shown in Figure 4. The attention mechanism is capable of learning information relative to the current input from sequence data. Control outputs generated by the net are expected to foresee the subsequent reference signals. Compared to implementing recurrent neural networks, applying attention layers for the trajectory input establishes relationships between the real-time control value and more distant desired signals. The attention module takes query, key, and value vectors as inputs. The queries contain current information, thus they are generated by a query net using the features provided by the input module. For every time step in the reference trajectory, an individual pair of key and value is produced using the key net and the value net. Then, given an input query $Q\in R^{d_{Q}}$, the attention score vector is calculated according to the key matrix $K\in R^{d_{K}\times n_{K}}$ as

(37) $$A=\mathrm{softmax}\left(\frac{Q^{⊤}K}{\sqrt{d_{Q}}}\right)$$

where $d_{K}$ is set to be equal to $d_{Q}$, and the softmax layer forces the sum of elements in the score vector to be one. Furthermore, the optimal control value should be irrelevant of states and reference signals before the current time step. Therefore, a time-dependent mask is implemented to the score vector, where the scores of past time steps are set to zero. Finally, the values $V\in R^{d_{V}\times n_{K}}$ are weighted by the attention score and summed to obtain the attention output

(38) $$o_{att}=V{\tilde{A}}^{⊤}$$

where $\tilde{A}$ is the masked score.

## 4. Simulation

In this section, we validate the proposed deep learning-based joint morphing and flight control method in simulated flight scenarios. The flight dynamics are given by (1) and (2). More specifically, the aerodynamic coefficients are obtained as [16]

(39) $$\begin{array}{cc} C_{L}\left(\alpha ,\xi ,\delta_{e}\right) & =C_{Lc}\left(\xi \right)+C_{L\alpha }\left(\xi \right)\alpha +C_{L\delta_{e}}\delta_{e}\\ C_{D}\left(\alpha ,\xi \right) & =C_{Dc}\left(\xi \right)+C_{D\alpha }\left(\xi \right)\alpha +C_{D\alpha^{2}}\left(\xi \right)\alpha^{2}\\ C_{M}\left(\alpha ,\xi ,\delta_{e}\right) & =C_{Mc}\left(\xi \right)+C_{M\alpha }\left(\xi \right)\alpha +C_{M\delta_{e}}\delta_{e} \end{array}$$

where the morphing-parameter-dependent aerodynamic derivatives are approximated using linear relationships as [19]

(40) $$C_{i}\left(\xi \right)=C_{i0}+C_{i1}\xi$$

Additionally, the relationship between the pitching moment of inertia and the morphing parameter is approximated as

(41) $$J_{y}=J_{0}+J_{1}\xi$$

**Figure 5:**
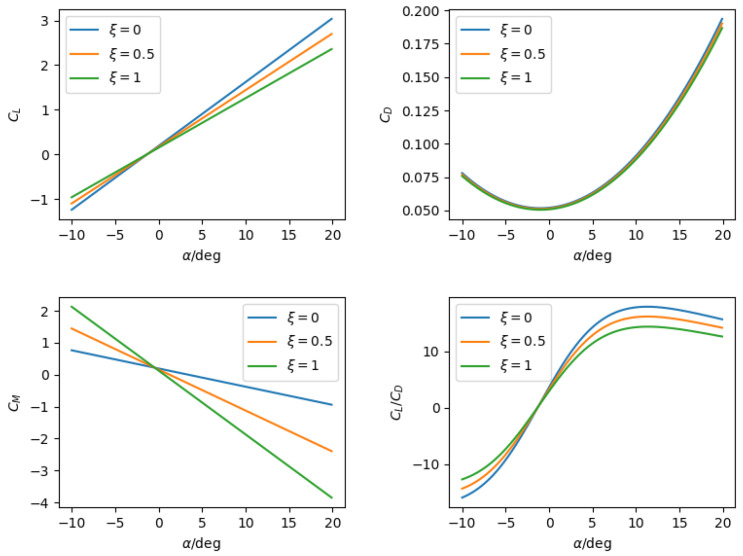
Variation in aerodynamic derivatives with respect to different morphing configurations.

Detailed values of the aircraft and aerodynamic parameters are given in Table 1 and Table 2, respectively. In Figure 5, the variation in aerodynamic derivatives with respect to different morphing configurations are illustrated. The simulation is comprised of two parts, which are the open loop solution comparison, and the learning-based control net test.

### 4.1. Open Loop Solution Comparison

**Figure 6:**
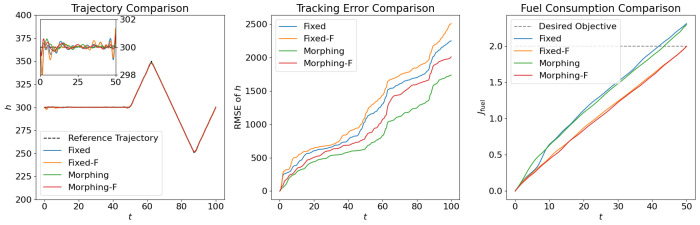
Comparison of the trajectories and objectives for open loop solutions with subordinate objectives of fuel consumption.

**Figure 7:**
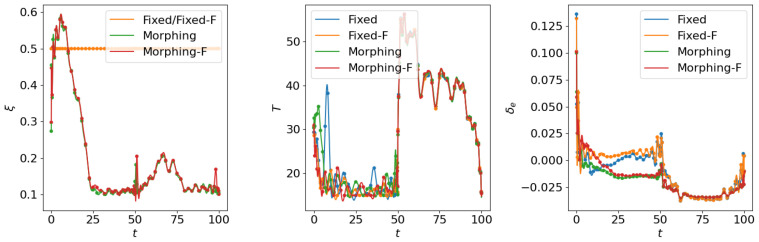
Comparison of the control inputs for open loop solutions with subordinate objectives of fuel consumption.

**Figure 8:**
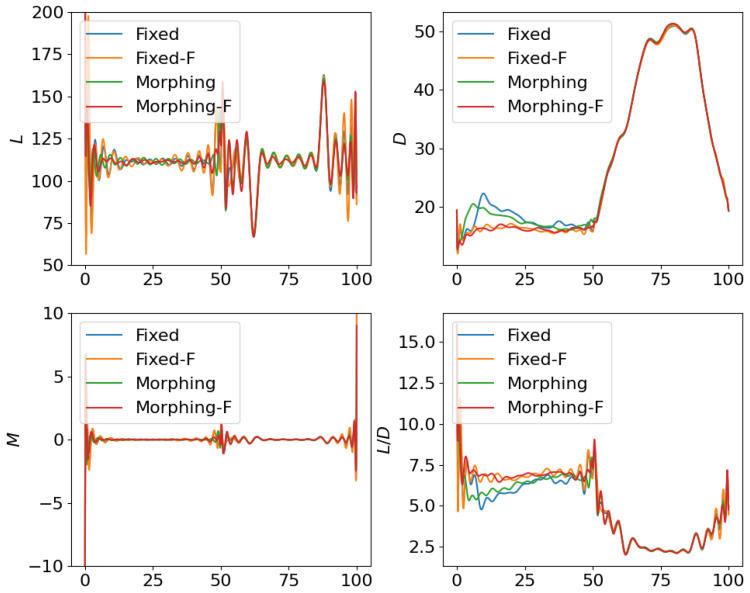
Comparison of the aerodynamic forces for open loop solutions with subordinate objectives of fuel consumption.

**Figure 9:**
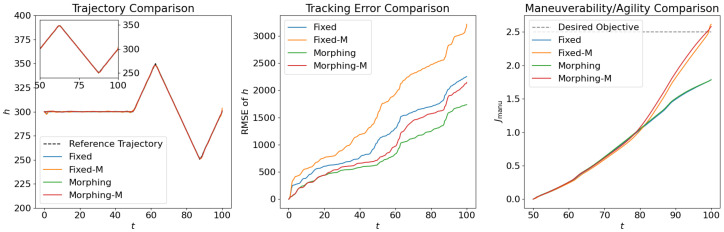
Comparison of the trajectories and objectives for open loop solutions with subordinate objectives of maneuverability and agility.

**Figure 10:**
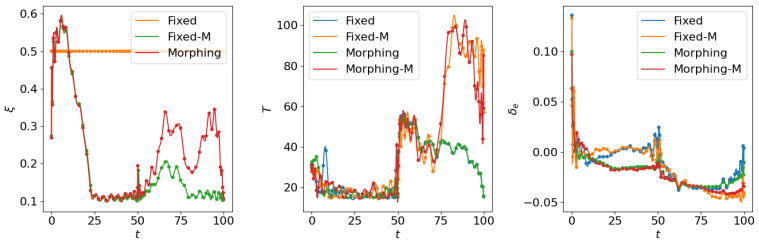
Comparison of the control inputs for open loop solutions with subordinate objectives of maneuverability and agility.

**Figure 11:**
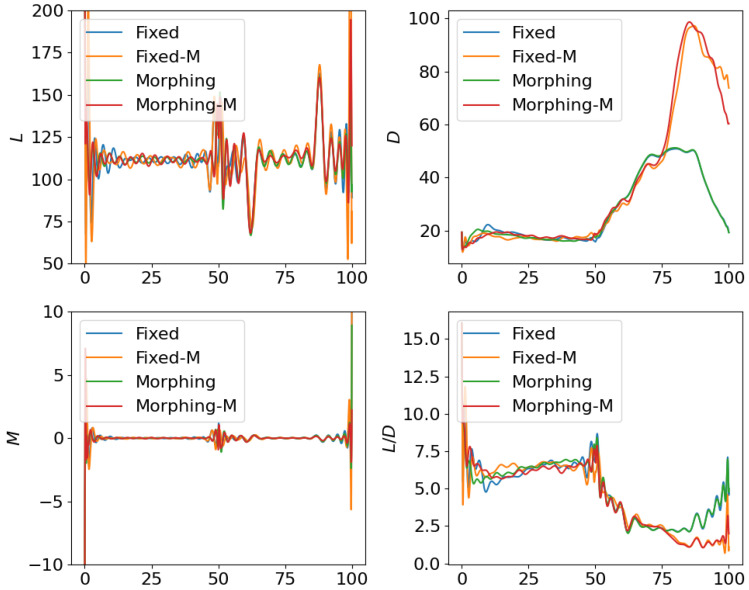
Comparison of the aerodynamic forces for open loop solutions with subordinate objectives of maneuverability and agility.

**Figure 12:**
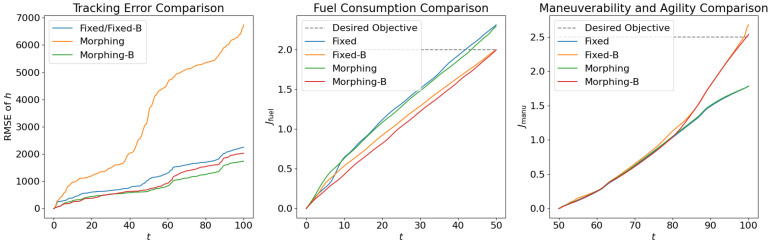
Comparison of the trajectories and objectives for open loop solutions with all subordinate objectives.

**Figure 13:**
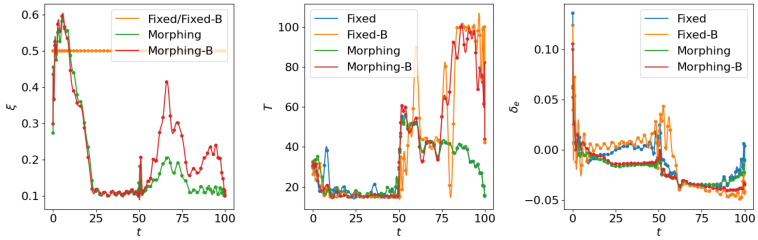
Comparison of the control inputs for open loop solutions with all subordinate objectives.

**Figure 14:**
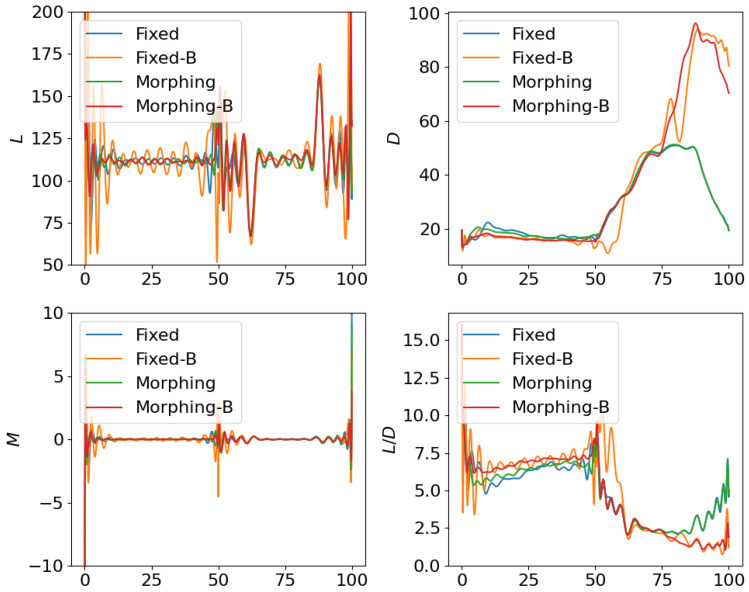
Comparison of the aerodynamic forces for open loop solutions with all subordinate objectives.

Firstly, we compare the optimization results obtained by the LGL pseudospectral method in Section 3.2 while the morphing capability is enabled or disabled. For a fixed wing aircraft, a constant constraint ξ=0.5 is imposed to the morphing parameter through the flight procedure with all other parameters kept the same. Simultaneously, we validate the effect of subordinate objectives that are relative to the fuel consumption, maneuverability, and agility. A typical reference trajectory is generated with constant-height cruising and a climbing–descending aggressive flight. The equation of the reference trajectory is given by

(42) $$h_{r}\left(t\right)=\left\{\begin{array}{cc} h_{\mathrm{const}},\; & t\in \left[0,t_{\mathrm{const}}\right]\\ h_{\mathrm{const}}+\frac{h_{\mathrm{upper}}-h_{\mathrm{const}}}{t_{\mathrm{upper}}-t_{\mathrm{const}}}\left(t-t_{\mathrm{const}}\right),\; & t\in \left[t_{\mathrm{const}},t_{\mathrm{upper}}\right]\\ h_{\mathrm{upper}}+\frac{h_{\mathrm{lower}}-h_{\mathrm{upper}}}{t_{\mathrm{lower}}-t_{\mathrm{upper}}}\left(t-t_{\mathrm{upper}}\right),\; & t\in \left[t_{\mathrm{upper}},t_{\mathrm{lower}}\right]\\ h_{\mathrm{lower}}+\frac{h_{\mathrm{const}}-h_{\mathrm{lower}}}{t_{f}-t_{\mathrm{lower}}}\left(t-t_{\mathrm{lower}}\right),\; & t\in \left[t_{\mathrm{lower}},t_{f}\right] \end{array}\right.$$

where time parameters $t_{\mathrm{const}}$, $t_{\mathrm{upper}}$, and $t_{\mathrm{lower}}$ and height parameters $h_{\mathrm{const}}$, $h_{\mathrm{upper}}$, and $h_{\mathrm{lower}}$ are tunable parameters, which determine the shape of the reference trajectory. In the open loop simulation, we set $t_{\mathrm{const}}=t_{f}/2$, $t_{\mathrm{upper}}=5t_{f}/8$, $t_{\mathrm{lower}}=7t_{f}/8$, $h_{\mathrm{const}}$ = 300 m, $h_{\mathrm{upper}}$ = 350 m, and $h_{\mathrm{lower}}$ = 250 m. The overall flight time is set as $t_{f}$ = 100 s. Besides the major tracking objectives, in the cruising procedure fuel consumption objectives (3) are considered, and in the aggressive flight procedure maneuverability and agility objectives (4) are involved. According to the problem transformation scheme in the previous section, we present three performance indices. The first is the major objective evaluating tracking performance, which is given by

(43) $$J_{\mathrm{major}}=\int_{T}\frac{∥h-h_{r}{∥}_{2}^{2}}{s_{\mathrm{major}}}dt$$

where the scale factor is set as $s_{\mathrm{major}}=10^{5}$. This is the first term in both the cost function (5) for cruise phase and the cost function (6) for aggressive flight phase. The second is the subordinate objective about fuel consumption in (3), which is given by

(44) $$J_{\mathrm{fuel}}=\int_{T_{c}}\frac{P}{s_{\mathrm{fuel}}}dt$$

where the scale factor is set as $s_{\mathrm{fuel}}=10^{5}$. This is the second term in the cost function (5) for the cruise phase. This term is transformed using the ϵ-constraint method with $\epsilon_{c}=0.04$, which means that the constraint $J_{\mathrm{fuel}}\leq 2.0$ is expected to be satisfied at the end of cruise flight phase. The third is the subordinate objective about maneuverability and agility in (4), which is given by

(45) $$J_{\mathrm{manu}}=\int_{T_{a}}\frac{∥p_{a}{∥}_{2}^{2}}{s_{\mathrm{manu}}}dt$$

where the scale factor is set as $s_{\mathrm{manu}}=10^{2}$. This is the negative of the second term in the cost function (6) for the aggressive flight phase. This term is transformed using the ϵ-constraint method with $\epsilon_{c}=-0.05$, which means that the constraint $J_{\mathrm{manu}}\geq 2.5$ is expected to be satisfied at the end of the aggressive flight phase. $N_{\mathrm{LGL}}^{\left(n\right)}=35$ points are collected as the LGL collocation points in the optimization. The simulation results are shown in Figure 6, Figure 7, Figure 8, Figure 9, Figure 10, Figure 11, Figure 12, Figure 13 and Figure 14.

From Figure 6, Figure 7 and Figure 8, we compare the results for fixed wing aircraft with only the tracking objective (Fixed), fixed wing aircraft with tracking and fuel objectives (Fixed-F), morphing aircraft with only the major objective (Morphing), and morphing aircraft with major and fuel objectives (Morphing-F). Comparisons of the trajectory and tracking error between Fixed/Morphing and Fixed-F/Morphing-F in Figure 6 show that when the configurations of objectives are same, the morphing aircraft achieves better tracking performance than the fixed wing aircraft. From the comparisons between Fixed/Fixed-F and Morphing/Morphing-F, it can be observed that with the subordinate fuel objectives, both fixed wing and morphing aircraft are able to reduce the fuel consumption to a desired level according to a given ϵ-constraint, while correlating with an increased tracking error. This validates the efficiency of the optimization for multiple objectives. The control inputs in Figure 7 show that the morphing parameter ξ varies throughout the flight, indicating that the aircraft adjusts its wing configuration to optimize aerodynamic performance and reduce fuel consumption. The thrust input *T* is also adjusted accordingly to support the fuel consumption optimization. Moreover, the aerodynamic forces in Figure 8 show that significantly lower lift-to-drag ratios are obtained for Fixed-F and Morphing-F, which contributes to a better fuel efficiency.

Similarly, we compare the optimization results with the subordinate objectives of maneuverability and agility in Figure 9, Figure 10 and Figure 11. The experiment configurations include fixed wing aircraft with only the tracking objective (Fixed), fixed wing aircraft with tracking and maneuverability/agility objectives (Fixed-M), morphing aircraft with only the major objective (Morphing), and morphing aircraft with major and maneuverability/agility objectives (Morphing-M). From the trajectories and objectives in Figure 9, analogous to the fuel consumption scenarios, the morphing aircraft demonstrates lower tracking error than the fixed wing aircraft when the maneuverability/agility objectives are involved. Desired maneuverability and agility indexes are achieved with the cost of a poorer tracking performance. In Figure 10, the morphing parameter varies significantly in the aggressive flight procedure, indicating that the aircraft continuously adjusts its wing configuration to pursue higher maneuverability and agility. This is further supported by the aerodynamic forces in Figure 11, where higher lift and drag forces are generated during aggressive maneuvers.

Following the test for individual subordinate objectives, we compare the optimization results with both subordinate objectives of fuel consumption at cruise phase and maneuverability/agility at aggressive flight phase. Corresponding results are shown in Figure 12, Figure 13 and Figure 14, where the settings include fixed wing aircraft with only the tracking objective (Fixed), fixed wing aircraft with tracking and both subordinate objectives (Fixed-B), morphing aircraft with only the major objective (Morphing), and morphing aircraft with major and both subordinate objectives (Morphing-B). Comparing the trajectories and major objectives in Figure 12, the morphing aircraft maintains a lower tracking error than the fixed wing aircraft in the multi-objective multi-phase scenario. Both of the subordinate objectives successfully achieve the desired performance, but the sacrifice of tracking performance is more significant than that in the single subordinate objective optimizations. Also, the lower lift-to-drag ratios in the cruise phase and higher lift and drag forces in the aggressive flight phase are observed simultaneously in Figure 14.

### 4.2. Control Net Validation

**Figure 15:**
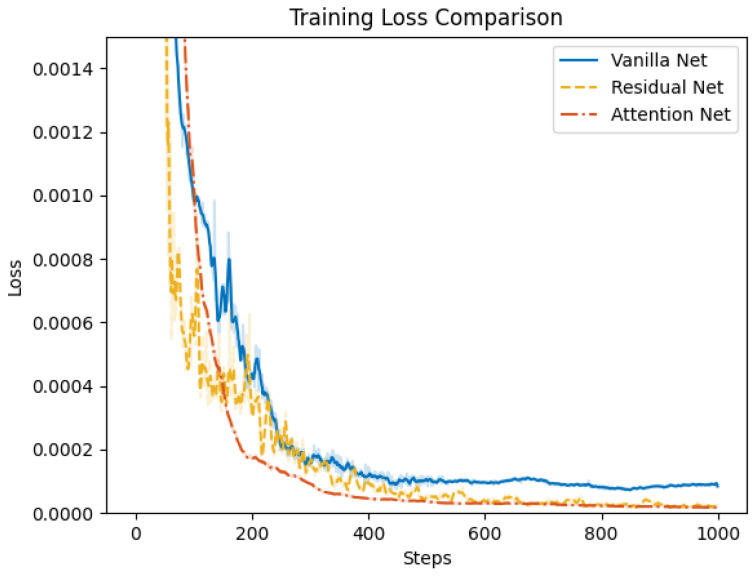
Comparison of training loss for vanilla net, residual net, and attention net.

**Figure 16:**
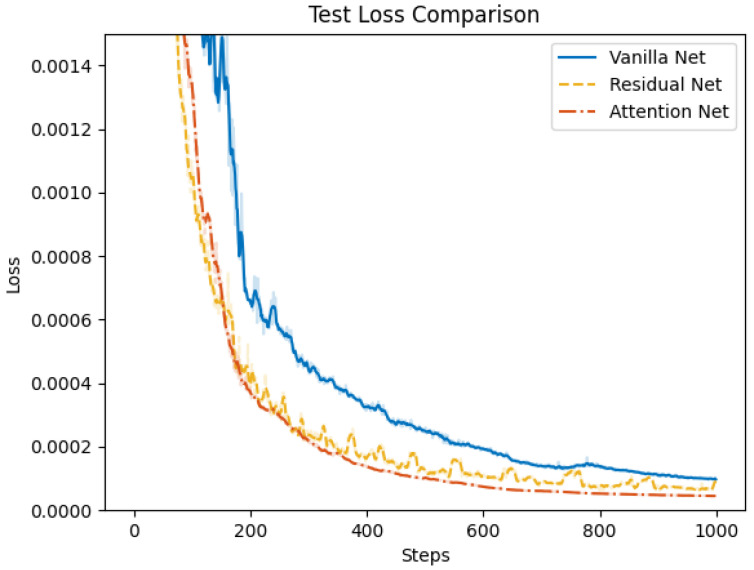
Comparison of test loss for vanilla net, residual net, and attention net.

Subsequently, we validate the proposed attention-based learning scheme for flight and morphing control. We conduct the training of the control net for the multi-objective multi-phase tracking mission, where the dataset is collected from the open loop solutions with both subordinate objectives. To illustrate the efficiency of individual modules in the control net, we compare the proposed attention net with two different structures. In vanilla net, both the attention mechanism and residual connections are removed. Fully connected layers are adopted to process the reference trajectory data, where the output is directly concatenated with the features from the input module. In residual net, skip connections are added to MLPs in the input and output modules. Training of the three networks is conducted with a stochastic gradient descent optimizer, where the learning rate is set to be 0.006 and the momentum is 0.9. Training loss and test loss during 1000 epochs of training are shown in Figure 15 and Figure 16, respectively. The results demonstrate that the attention net outperforms the vanilla net and residual net in terms of both training and test loss. Additionally, the proposed attention net achieves a faster convergence. This indicates that the attention mechanism is capable of extracting more informative features from the reference trajectory data and improving the control performance.

Finally, we incorporate the networks into the flight and morphing procedure. Besides the attention-based control net for both subordinate objectives, we train a controller for only the major objective to comprehensively demonstrate the efficiency of the morphing mechanism and learning control networks. The learning-based controllers with and without subordinate objectives are denoted as Net-Morphing-B and Net-Morphing, which are compared with the offline open loop optimized solutions Fixed, Fixed-B, Morphing, and Morphing-B.

**Figure 17:**
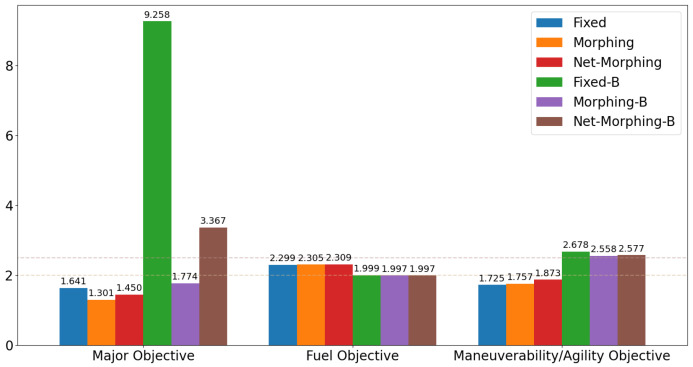
Comparison of normalized objectives. Dotted lines indicate the desired values of the subordinate objectives.

The results are shown in Figure 17. With regard to the tracking performance, it is observed that open loop solutions for morphing aircraft obtain lowest tracking errors, which is reasonable since they are optimized offline and serve as the training data for the control net. Net-Morphing/Net-Morphing-B solutions achieve slightly higher tracking errors than Morphing/Morphing-B solutions, while they are still better than the fixed wing aircraft. Such results validate the approximation capability of the proposed control net, and further demonstrate the efficiency of the morphing ability in enhancing the aircraft’s tracking performance. Additionally, in this case the fuel consumption is desired to be lower than 2.0 and the maneuverability/agility objective is desired to be larger than 2.5. From the results in Figure 17, we can see that the proposed learning control scheme successfully satisfies the ϵ-constraints and accomplishes the multi-objective optimal control.

## 5. Discussion

### 5.1. Considerations for Real-World Deployment

While this work focuses on the theoretical framework and simulation-based validation of the learning-based joint morphing and flight control system, several considerations remain for the real-world deployment of the proposed method on actual avian-inspired aircraft. Potential challenges and corresponding approaches in areas such as sensor integration, real-time computation, actuator dynamics, robustness, and safety are discussed below.

A comprehensive suite of avionics sensors is necessary to acquire the accurate values of desired system states. In this work, the fundamental states—including longitudinal velocity, angle of attack, pitch rate, pitch angle, and altitude—can typically be measured using a synthetic Air Data System. Such a system integrates data from the Global Positioning System (GPS), inertial measurement units (IMU), the pitot-static system, and angle-of-attack vanes. Nonlinear estimation techniques, including the Extended Kalman Filter (EKF) or particle filters, are commonly employed to fuse information from these diverse sensors and mitigate the impact of measurement noise. Considering the aircraft’s morphing capabilities, monitoring the morphing parameters is essential for a reliable controller. For wing sweep configurations, sensors like rotary variable differential transformers (RVDTs) can provide real-time sweep angle measurements. For other morphing configurations, such as wing span or camber morphing, airfoil shapes can be determined using sensors based on optical fibers, piezoelectric materials, and electro-active polymers [59]. The time-varying shape configuration and the multi-phase mission with different tasks result in non-persistent state transition dynamics in the Bayesian filtering framework, which brings challenges for state estimation and sensor integration. To address this, an Interacting Multiple Model (IMM) framework, implementing multiple local nonlinear filters tailored to different shape configurations, offers a promising approach to facilitate real-time flight mode identification and provide reliable information for the learning-based controller.

Concerning real-time computation, our simulation results show that the neural network controller with attention modules, running on an NVIDIA GeForce RTX 4060 GPU, achieves an inference time of 2.72 ms per control cycle. This demonstrates the potential for high-frequency control. However, implementing such a computationally intensive network on an actual avian-inspired aircraft requires careful consideration of onboard computing capabilities. Given the typical size, weight, and power (SWaP) constraints of aircraft, the direct use of high-performance GPUs is limited. For deep learning-aided morphing aircraft, it is more suitable to deploy embedded, reduced-board hardware, such as the Jetson Nano or Google Coral, coupled with additional computation-intensive payload modules for neural network acceleration [60,61,62,63]. These platforms offer a balance between computational power and energy efficiency. To bridge the gap between simulation performance and real-time onboard execution, neural network optimization techniques, including model pruning, quantization, and knowledge distillation, can be employed to reduce model complexity and improve inference speed.

Electromechanical actuators and hydraulic cylinders are common choices for achieving wing sweep morphing in aircraft. Electromechanical actuators offer precision and controllability, while hydraulic systems can provide the high forces necessary for larger wings. During the sweep morphing process, frequently encountered control issues with these actuators include backlash and friction. Backlash, which causes lost motion in mechanical linkages, negatively impacts speed and position control accuracy. Extensive research exists on backlash compensation techniques. For the control framework proposed in this work, this effect could be mitigated by cascading an additional nonlinear controller between the morphing parameter signal and the actuator input, potentially incorporating a disturbance observer to compensate for the control gap. Friction, both static and dynamic, can lead to jerky, reciprocating motions and reduced control precision. Similarly, constructing an extra compensation controller based on friction models, such as the LuGre model and the Coulomb–viscous friction model [64], offers a viable approach to address friction issues. With the advancement of smart materials, actuators based on shape memory alloys (SMAs) and shape memory polymers (SMPs) can facilitate more complex morphing mechanisms. A crucial dynamic characteristic of shape memory actuators is their hysteresis. Consequently, additional design of actuator controllers employing models like the Preisach model and the Krasnosel’skii–Pokrovskii model becomes necessary to achieve accurate control [65].

Ensuring the robustness of the proposed learning-based control framework to real-world uncertainties and disturbances is critical for the practical implementation. Although our simulation environment provides controlled scenarios, actual flight will inevitably present unmodeled dynamics, sensor noise, wind gusts, and potential variations in aircraft parameters caused by manufacturing tolerances or environmental factors. Future work should investigate several topics to enhance the robustness and safety of the neural network controller. A promising approach is to augment the training data with additional uncertainties and disturbances, enabling the neural network to learn inherently more robust control policies. Exploring learning techniques including adversarial training with robustness-specific fine-tuning can also make the controller less sensitive to the perturbations in the input data [66]. Moreover, integrating robust control methods, such as H*∞* control or sliding mode control, with the proposed learning framework will provide theoretical guarantees on stability and performance with bounded uncertainties. Finally, extensive flight simulation across a spectrum of realistic and extreme conditions, along with potential hardware validation, will be essential to thoroughly evaluate and enhance the robustness of the proposed neural network controller before real-world deployment.

### 5.2. Extending the Framework to More Complex Flight Scenarios

In this work, we consider the flight scenario composed of cruise flight and aggressive flight. With consideration of tracking error as a primary objective, we prioritize fuel efficiency for cruise flight and maneuverability/agility for aggressive flight. Based on this, a multi-phase multi-objective optimal control problem is formulated. More intricate flight situations can be addressed by developing specific objective functions within the proposed optimal control problem. This inherent capability to augment flight phases with task-tailored performance metrics allows for the extension of the proposed learning control framework to more complex flight scenarios.

For instance, in obstacle avoidance tasks, a typical Sense and Avoid (S&A) system for aircraft integrates sensing hardware, a decision mechanism, a path planner, and a flight controller [67]. With the successful detection of obstacles and decision for avoidance, the flight controller can track a replanned path with obstacle constraints directly, or incorporate the avoidance objectives. These objectives can be formulated as collision costs with penalty terms for the proximity to detected obstacles, which will increase significantly as the distance between aircraft and identified obstacle fields decreases [68]. The epsilon constraint applied to this obstacle avoidance objective determines the priority of safety over other objectives.

Although formation flying missions involve coordinating aircraft swarms, precise control of individual aircraft remains crucial, particularly within distributed formation control frameworks [69]. A common objective function for formation flying is minimizing the tracking error between each aircraft and its reference trajectory, as defined by the formation generator [70]. Furthermore, obstacle avoidance objectives must also be considered to prevent collisions between aircraft within the formation. In the virtual structure approach, objectives are designed to maintain specific relative positions and orientations with respect to a predefined virtual point on a virtual rigid body. For behavior-based control strategies, specific objectives can be defined according to basic rules such as separation, aggregation, and speed matching. Within a consensus-based framework, the objective is typically designed to achieve state consistency among the agents [69].

Flight control in highly dynamic settings presents significant challenges, especially when disturbances like gusty winds and turbulent air impact the morphing procedure of morphing aircraft. Potential extensions of the proposed framework to such scenarios include designing objective functions that enhance robustness and minimize the impact of these disturbances on both morphing and flight control. Additionally, control policies that minimize task completion time to reduce risk, or maximize the observability of flight dynamics to improve sensing performance, can be considered. Moreover, incorporating state observers and robust control techniques can further enhance performance for operations in highly dynamic scenarios [15,22].

### 5.3. Neural Network Architecture and Learning Mechanism

In this work, we introduce a neural network controller designed to learn a feedback control law for the multi-objective morphing and flight control task. Our control network employs MLP modules with residual connections for effective state feature extraction and temporal masked attention modules for processing sequential reference trajectory data. This section discusses the potential for further enhancing the learning mechanism, focusing on three aspects: more efficient data generation strategies, a comparative analysis with alternative temporal neural network architectures, and a discussion on the relationship with reinforcement learning methods.

The quality and diversity of the training data are crucial for the performance of the learning control method. In the simulations, the training dataset is generated by solving a series of open loop optimal control problems using a pseudospectral method, with variations introduced by different parameters of the predefined reference trajectory. Subsequently, the network explores a range of flight scenarios in the state–action space. However, more effective data generation strategies will significantly enhance the learning process. A promising approach is to implement a neural network warm start mechanism for open loop optimal trajectory generation [71]. Solving many NLP problems arising from the pseudospectral discretization across various reference trajectories from is computationally expensive. However, a neural network controller, even in its early stages of training, can provide suboptimal initial guesses for these NLP problems. Therefore, the NLP solver can adopt the suboptimal trajectories generated by the neural network controller as a warm start, to achieve faster convergence and generate a richer and more diverse set of training trajectories that better cover the region of interest. Conducting iterative data generation and supervised training could significantly improve the learning efficiency. Additionally, explicitly enlarging the coverage of the state–action space during the flight procedure will lead to more informative data. The methods include injecting noises and disturbances into the system dynamics and actively generating trajectories that explore less-visited regions. Future works should systematically investigate these more effective data generation strategies to create richer and more informative training datasets, and develop a more efficient, robust, and generalizable learning-based control framework for avian-inspired morphing aircraft.

In the proposed learning framework, a temporal masked attention module is employed to extract relevant information from the reference trajectories. While recurrent neural networks (RNNs), such as GRUs and LSTMs, are also well suited for processing sequential data, the choice of attention over RNNs in this work is mainly motivated by three key aspects. Firstly, attention mechanisms inherently possess the capability to selectively focus on the most relevant parts of the input sequence, while in traditional RNN structures more recent inputs are given greater weight. In the context of our multi-phase flight missions, the controller is expected to anticipate not only immediate reference signals but also potentially distant trajectory points that convey information about various tasks. Adopting attention modules could help the controller focus the most relevant information in the future trajectory and adjust the control policy accordingly. Secondly, during the online flight procedure, namely the inference phase, the reference trajectory from the current time step to the end time step must be processed every control cycle to capture the relationship between the current state and future tasks. While the attention network can process the entire time sequence in parallel, an RNN with a comparable parameter scale needs to process the sequence step by step. This sequential processing can significantly increase the inference time, which is a critical factor for real-time flight as discussed in the previous section. Finally, the attention mechanism is preferred for its potentially more efficient training procedure. Training RNNs requires backpropagation through time (BPTT), which can be difficult to parallelize and often leads to slower training. Furthermore, deep RNNs processing long sequences suffer from the vanishing and exploding gradient problems. In contrast, the parallel processing and direct connections between distant parts of the sequence in attention networks can contribute to a more efficient and stable training process. In future work, a comprehensive ablation study should be conducted where different types of RNN modules with a similar parameter scale replace the attention module for processing reference trajectories. The tracking performance, training convergence speed, and inference time of these alternative architectures will be compared.

The proposed attention-based morphing and flight control method employs supervised learning to derive the control policy from offline optimal solutions. Alternative approaches like Adaptive Dynamic Programming (ADP)/Reinforcement Learning (RL) have also shown significant capability for solving complex optimal control problems. For the dexterous manipulation tasks with high-dimensional environments, RL methods combining generative adversarial architecture demonstrate the ability of RL to learn complex control policies and achieve impressive manipulation skills [72]. Region-based approximation methods enhance ADP for systems with large state spaces by partitioning the domain, leading to improved value function approximation and closed loop performance [73]. For general nonlinear systems with unknown dynamics, the zero-sum two-player game is constructed using Q-learning-based approach to solve the Hamilton–Jacobi–Isaacs (HJI) equation, leading to a robust control framework [74]. While ADP/RL offers powerful techniques for learning the optimal controller, our proposed method provides a complementary approach with its own set of advantages. In contrast to the acquisition of data from trial-based exploration in RL settings, the proposed supervised learning framework leverages offline optimal solutions generated from task-specific open loop optimal control problems and pseudospectral discretization. This strategy contributes to a more sample-efficient and interpretable learning procedure, since training trajectories can be precisely defined through adjustments to the offline optimization. Moreover, given the availability of high-quality open loop solutions from prior nonlinear optimizations, training a supervised learning controller can be more computationally efficient than RL, as it eliminates the requirement for online interaction with the environment. Future work will investigate the application of ADP/RL methods for integrated morphing and flight control and explore combining attention-based learning control with RL techniques to develop more robust and efficient control frameworks for general multi-objective multi-phase optimal control problems.

### 5.4. Comparison with Recent Advances in Integrated Morphing and Flight Control

In this work, a learning-based control framework is proposed for integrated morphing and flight control in multi-objective, multi-phase flight scenarios. To provide a more comprehensive context for our proposed method and to elucidate its distinctive benefits, a comparison with recent advancements in morphing aircraft control that concurrently incorporate morphing and flight control is discussed.

In [75], a co-design optimization method for morphing topology and flight control is presented. This method designs a morphing drone with shapes determined by joint configurations to accomplish a flight task that balances energy consumption and completion time within a specific flight scenario. An optimization problem is formulated wherein joint configurations and propeller thrust are the decision variables, and the optimal morphing topology and control inputs are obtained by minimizing a cost function that combines time and energy. A cooperative game control method with safe reinforcement learning (RL) is proposed in [76]. They formulate an affine nonlinear tracking error model where the wing deformation parameter acts as an additional input. A safe RL-based adaptive cooperative game solution is developed for the coordinated optimal control problem, which minimizes the tracking error. In [77], an integrated decision-making and control framework is introduced for morphing flight vehicles during the glide phase. This framework designs a comprehensive performance index that incorporates the lift-to-drag ratio and attitude control effect. By combining a deep deterministic policy gradient (DDPG)-based dynamic programming solver for morphing strategy with an additional ADP-based attitude controller, the glide range is maximized. In [78], a four-wing variable-sweep aircraft is considered, where the control inputs include sweep angles and thrust magnitude. For a reference trajectory tracking mission, they establish a reinforcement learning environment with an integrated reward that accounts for tracking performance and flight stability. An incremental deep deterministic policy gradient (IDDPG) method, enhanced with LSTM networks for obstacle avoidance, is used to determine the integrated control policy.

The aforementioned cutting-edge techniques provide insightful contributions to integrated morphing and flight control. The distinctive benefits of the proposed methodology in this work are mainly in three aspects.

Firstly, while the aforementioned methods focus on specific morphing configurations and flight scenarios, our work develops a general multi-objective, multi-phase optimal control framework. Complex and time-varying tasks can be incorporated into our framework by designing corresponding objective functions, demonstrating the flexibility and adaptability of the proposed method. Moreover, compared with the weighted sum formulation of multiple objectives used in the mentioned works, our method employs the ϵ-constraint method to provide a flexible guarantee for satisfying subordinate objectives. Secondly, compared with the co-design optimization method [75], our method provides a learning scheme to generate feedback online control laws from offline optimization results. Thirdly, compared with the RL-based methods [76,77,78], our framework combines offline open loop solutions with an online learning controller. The offline optimal solutions generated by the pseudospectral method provide high-quality training data. With such training trajectories, our supervised learning scheme avoids the cumbersome exploration process inherent in RL and achieves a more sample-efficient and interpretable learning procedure. Furthermore, the incorporation of an attention mechanism helps the controller extract information from the most relevant parts of the sequential data, which is beneficial for multi-phase flight missions. In future work, we will further explore the incorporation of advanced control methods and learning techniques in our integrated morphing and flight control framework.

## 6. Conclusions

This paper presents an attention-based learning framework for avian-inspired morphing aircraft, where the morphing strategy is integrated into the flight control. A multi-objective, multi-phase optimal control problem is formulated to model comprehensive flight missions with diverse tasks, specifically considering a wing sweep morphing aircraft performing aggressive-cruise synthesized scenarios. The auxiliary problem is constructed using ϵ-constraint and augment state methods to establish the connection between open loop solutions and the feedback control rule. Then, the auxiliary optimal control scheme is discretized with specific LGL pseudospectral methods, and open loop solutions are generated from the resulting NLP problem. Subsequently, a neural network-based feedback controller is trained using the generated offline optimal solutions, incorporating a temporal masked attention mechanism to effectively process sequential data. Simulation results demonstrate that the morphing mechanism improves tracking performance and facilitates the satisfaction of subordinate objectives, and the effectiveness of the proposed learning control framework is validated. Future work will focus on extending the framework to more complex scenarios, exploring more robust data generation methodologies, and developing more efficient network architectures for morphing control.

## Figures and Tables

**Table 1:** Values of aircraft parameters.

Parameter	Value	Parameter	Value
*m*	11.4 kg	*g*	9.8 m/s^2^
$\psi_{\max}$	40 deg	ρ	1.29 kg/m^3^
*S*	0.84 m^2^	$\bar{c}$	0.288 m

**Table 2:** Values of aerodynamic parameters.

Parameter	Value	Parameter	Value
$C_{Lc0}$	0.19	$C_{Lc1}$	−0.04
$C_{L\alpha 0}$	0.143	$C_{L\alpha 1}$	−0.032
$C_{Dc0}$	0.052	$C_{Dc1}$	−0.0012
$C_{D\alpha 0}$	0.00065	$C_{D\alpha 1}$	−0.000026
$C_{D\alpha^{2}0}$	0.000325	$C_{D\alpha^{2}1}$	−0.000013
$C_{Mc0}$	0.195	$C_{Mc1}$	−0.065
$C_{M\alpha 0}$	−0.057	$C_{M\alpha 1}$	−0.057
$C_{L\delta_{e}}$	−0.02	$C_{M\delta_{e}}$	0.125
$J_{0}$	3.04	$J_{1}$	0.6

## Data Availability

Dataset available on request from the authors.

## Abbreviations

The following abbreviations are used in this manuscript:

LPV	Linear Parameter Varying
RBF	Radial Basis Function
RNN	Recurrent Neural Network
MLP	Multilayer Perceptron
LGL	Legendre–Gauss–Lobatto
NLP	Nonlinear Programming
SGD	Stochastic Gradient Descent
